# Surrogate modelling of heartbeat events for improved J-peak detection in BCG using deep learning

**DOI:** 10.3389/fnetp.2024.1425871

**Published:** 2024-07-19

**Authors:** Christoph Schranz, Christina Halmich, Sebastian Mayr, Dominik P. J. Heib

**Affiliations:** ^1^ Human Motion Analytics, Salzburg Research Forschungsgesellschaft mbH, Salzburg, Austria; ^2^ Department of Artificial Intelligence and Human Interfaces, University of Salzburg, Salzburg, Austria; ^3^ Institut Proschlaf, Salzburg, Austria

**Keywords:** ballistocardiogram, ballistocardiography, J-peak, heartrate estimation, event detection, peak detection, deep learning, ResNet

## Abstract

Sleep, or the lack thereof, has far-reaching consequences on many aspects of human physiology, cognitive performance, and emotional wellbeing. To ensure undisturbed sleep monitoring, unobtrusive measurements such as ballistocardiogram (BCG) are essential for sustained, real-world data acquisition. Current analysis of BCG data during sleep remains challenging, mainly due to low signal-to-noise ratio, physical movements, as well as high inter- and intra-individual variability. To overcome these challenges, this work proposes a novel approach to improve J-peak extraction from BCG measurements using a supervised deep learning setup. The proposed method consists of the modeling of the discrete reference heartbeat events with a symmetric and continuous kernel-function, referred to as surrogate signal. Deep learning models approximate this surrogate signal from which the target heartbeats are detected. The proposed method with various surrogate signals is compared and evaluated with state-of-the-art methods from both signal processing and machine learning approaches. The BCG dataset was collected over 17 nights using inertial measurement units (IMUs) embedded in a mattress, together with an ECG for reference heartbeats, for a total of 134 h. Moreover, we apply for the first time an evaluation metric specialized for the comparison of event-based time series to assess the quality of heartbeat detection. The results show that the proposed approach demonstrates superior accuracy in heartbeat estimation compared to existing approaches, with an MAE (mean absolute error) of 1.1 s in 64-s windows and 1.38 s in 8-s windows. Furthermore, it is shown that our novel approach outperforms current methods in detecting the location of heartbeats across various evaluation metrics. To the best of our knowledge, this is the first approach to encode temporal events using kernels and the first systematic comparison of various event encodings for event detection using a regression-based sequence-to-sequence model.

## 1 Introduction

Sleep has a profound influence on the physical and mental functioning of the following day, especially when it is lacking. Restful sleep promotes physical regeneration (Jakowski et al., 2023) and the performance of the immune system (Garbarino et al., 2021), as well as cognitive performance (Brownlow et al., 2020), motor dexterity (Craven et al., 2022) and emotional stability (Tomaso et al., 2021). Disturbed sleep can lead to serious chronic health problems such as cardiovascular disease, endocrinological dysregulation, and a range of psychological impairments (Itani et al., 2017). To elucidate the dynamics and interactions of sleep, a comprehensive understanding of sleep patterns and stages is essential. The gold standard for objectively measuring sleep is polysomnography (PSG), which integrates electroencephalography, electromyography, and electrooculography. However, PSG recordings are time-consuming, the equipment is costly, and trained personnel are required to ensure sufficient signal quality. These drawbacks limit the sample sizes of PSG studies and render them unsuitable for longitudinal studies. Recent trends in sleep research indicate that high-accuracy estimations of sleep stage fluctuations can be derived from variations in signals such as inter-beat interval (IBI) time-series (kranzinger et al., 2023) or alterations in respiratory effort over time when analyzed with machine learning models.

Today, IBI time-series can be accurately recorded using inexpensive consumer devices, making inter-beat intervals a promising signal for large-scale sleep studies aimed at gaining new insights into sleep. State-of-the-art sensors for measuring heartbeats can be categorized into on-body (wearables) and off-body (contactless) solutions. On-body systems include devices directly attached to the body, such as electrocardiography and photoplethysmography to acquire electrocardiograms (ECG) and photoplethysmograms (PPG). PPG wearables, typically mounted on the wrist, arm, or earlobe, measure heartbeats by detecting periodic changes in the optical reflection of emitted light caused by blood pulses under the skin.

Contactless sensor systems mainly involve camera-based systems as well as ballistocardiography. Camera-based systems sense minor periodic changes in the skin color caused by blood pulses. Ballistocardiography is a sensor system that measures subtle accelerations of the human body, including cardiovascular and respiratory activity, that are plotted in ballistocardiograms (BCG). The heartbeat events in BCG are referred to as J-peaks and are caused by the contraction of the heart which results in the ejection of blood into the aortic arch where the direction of flow is changed, creating a momentum (Giovangrandi et al., 2011). Therefore, the systolic J-peak occurs after the electrical trigger, i.e. the R-peak, as accessed from an ECG. These events also occur closer to the heart and are sharper in their waveform than camera-based solutions or PPG, where the monitored pulses are measured at the skin or wrist.

**FIGURE 1:**
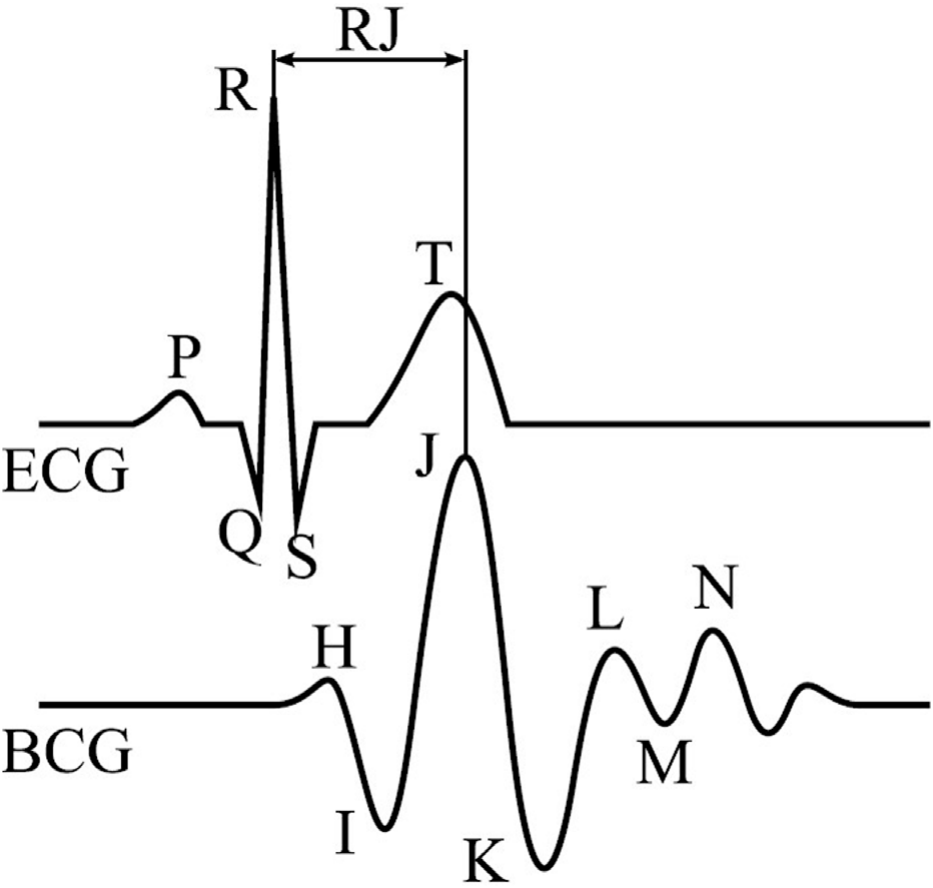
Single beat of an ECG (top) and BCG (bottom) with their annotated main waves and RJ-interval (Gomez-Clapers et al., 2014).

The delay between the electrical activation of the heart muscle to the greatest vertical force as measured by a BCG-system is referred to as RJ-interval. A schematic ECG and BCG with their corresponding QRS- and IJK-complexes are illustrated in Figure 1. According to the literature, the RJ-intervals vary typically between 180 ms and 240 ms and change slowly over time (Casanella et al., 2012). J-peaks in BCG occur closer to the heart and are sharper in its waveform than PPG, which measures pulses at the skin or wrist.

The inter-subject variability is caused by different causes such as body mass, heart size, body placement relative to the fixed sensor position, body alignment, and also the physiological state of the subject. Additionally, the BCG also depends on the used sensor type and setup Sadek and Abdulrazak (2021). Some activities, such as paced respiration, can induce hemodynamic changes that affect the RJ-interval by 150 ms–300 ms (Casanella et al., 2012; Gomez-Clapers et al., 2014).

Ballistocardiogram can be implemented using different sensor technologies. The most commonly used are inertial measurement units (IMU) (Cathelain et al., 2020), electromechanical films (Sadek and Abdulrazak, 2021), or piezoelectric (Zhou et al., 2021; Liu et al., 2022), hydraulic- (Heise and Skubic, 2010) or pneumatic- (Pröll et al., 2019) pressure sensors. In each case, the sensor is integrated into the mattress, pillow, mat or chair underneath the person, allowing for an unobtrusive measurement. This type of unobtrusive measurement in particular offers a seamless recording of sleep data over several weeks at home without the need for a sleep laboratory, as no sensors or wearables need to be actively applied or activated. Data acquisition is activated simply by lying in bed.

Off-body measurement systems therefore offer a more elegant and unobtrusive means of recording physiological information over extended periods, as they typically require minimal interaction with the recording device, thereby reducing distress and potential user resistance associated with long-term use of wearables. However, as the indirect measurement leads to a decreased signal-to-noise ratio, detecting individual heartbeats from contactless sensors is significantly more challenging compared to wearables. This limitation of contactless devices is critical, as the accuracy of automatic sleep stage classification based on IBI time-series depends on the temporal precision of the captured IBIs. Therefore, advancements in heartbeat extraction from BCG are crucial.

Given the suitability of ballistocardiography for unobtrusive long-term sleep measurement, and the availability of improved machine learning algorithms and computational resources, there is an increasing amount of research focused on heartbeat extraction from BCG. Research has shown that J-peaks can be used to predict the subject’s sleep stages and therefore sleep quality (Kranzinger et al., 2023). This work proposes a novel approach to model heartbeat events as a continuous signal, thereby improving the accuracy of heartbeat extraction within a supervised deep learning framework. The primary objective is to evaluate various heartbeat event representations in combination with different deep learning network architectures for J-peak detection in BCG signals and to compare them with existing methods.

Section 2 introduces the state-of-the-art methods and reasons why machine learning approaches might offer advantages in overcoming existing limitations of current contact-less methods. Section 3 provides a formal introduction to the problem from a theoretical perspective, and Section 4 presents the proposed method and evaluation of the methods. In Section 5 the results of the method comparison are presented. Finally, the findings are discussed in Section 6 and concluded in Section 7.

## 2 Related work

The classical approach for the detection of heartbeats in ballistocardiogram (BCG) is the Pan-Tompkins algorithm (Pan and Tompkins, 1985). This algorithm was initially developed for ECG and is based on classical signal processing techniques, such as low and high-pass filtering, derivates, functional mappings, and averaging. Using the thereby processed signal, a peak detection algorithm is applied to detect the characteristic R-peak of the ECG. However, the low signal-to-noise ratio of BCG data limits the detection of heartbeats using the same approach. Hence, the Pan-Tompkins algorithm was adapted for the application on BCG data. Most solutions employ a bandpass filter as the initial processing step, with a recommended system bandwidth ranging from 1.5 Hz to 22.5 Hz. This frequency band encompasses all relevant cardiovascular signals while filtering out respiratory activity and movements (Gomez-Clapers et al., 2014). For instance, Pröll et al. (2019), applied the following sequential processing steps: bandpass filter, cubic function, low-pass filter, second order derivate, absolute value function, and low-pass filter (Pröll et al., 2019). This processing pipeline transforms the raw BCG signal into a signal that exhibits the characteristic J-peak of the BCG more significantly. A subsequent peak detection identifies the IJK-complex that is analogous to the QRS-complex in ECG.

Other classical signal processing approaches apply wavelet transformations, template matching, or signal envelopes (Pino et al., 2017; Sadek and Abdulrazak, 2021). Additionally, some approaches apply methods in the frequency domain (Brüser et al., 2011). Analogously, classical signal processing approaches for R-peak extraction in noisy ECG and PPG measurements are based on a similar combination of algorithms (Nguyen et al., 2019; Yun et al., (2022)).

As pointed out in Section 1, the substantial inter- and intrasubject variability of BCG as well as the low signal-to-noise ratio remain major challenges of the J-peak extraction in BCG measurements. In order to address these challenges, neural networks can be used that are effective in capturing the variability of BCG within a data-driven supervised machine learning setting. Pröll et al. (2021); Sadek and Abdulrazak (2021) demonstrated that their deep learning approach, using a combination of convolutional neural networks (CNN) and recurrent network layers (LSTM and GRU) of different sizes, has improved the accuracy of estimating the mean heart rate within 8 s epochs by more than 50% in terms of MAE compared to five state-of-the-art digital signal processing approaches (from 4.24 to 2.07). This approach estimates the heart rate from a BCG signal and compares it with a reference heart rate as accessed from an ECG. Other approaches apply deep learning models to approximate a signal with characteristic J-peaks. For example, Cathelain et al. (2020); Zhou et al. (2021) have applied the U-net architecture and (Liu et al., 2022) a combination of Residual Networks (ResNet) and long-short term memory (LSTM). Most of these methods, both based on traditional digital signal processing and neural networks, have in common that they process the input BCG data to emphasize the J-peaks. Moreover, for all deep learning models found in literature review, the discrete J-peak-events are represented as a time-series encoded with a binary masking. This binary masking, however, may lead to inaccurate peak detection, as further discussed in Section 3.

In this work, a method based on deep learning is proposed, which transforms a BCG measurement into a one-dimensional time-series, from which the discrete heartbeat events can be detected more precisely. The objective of the work is to compare the effect of different heartbeat encodings on J-peak detection accuracy using a fixed neural network architecture, and to compare the proposed method against state-of-the-art approaches. Thereby, we investigate improved encodings of heartbeat events in order to facilitate an optimized J-peak detection.

## 3 Problem formulation

The objective of J-peak detection in BCG is to estimate the timestamps of heartbeat events P in time-series data X . Consequently, a J-peak detector implements an algorithm that maps X to P . The input BCG are single- or multichannel measurements, which are represented as $X\in R^{n\times k}$ with k being the number of channels and n the number of equidistantly sampled measurement points. In order to detect heartbeats present in X , each deep learning approach for J-peak detection mentioned in Section 2 models the J-peaks, a set of event timestamps P={p∈R} , with a binary event-hot encoding in the corresponding target time-series $y\in R^{n}$ . Additionally, a small area of interest around the peak with a width of τ may also be encoded with one. In the context of this work, y is referred to as surrogate signal. The majority of machine learning models M employed for this task are implemented as sequence-to-sequence models, which learn the following mapping Eq. 1:

M:X↦y (1)

Given that an ideal model M is unlikely to exist, the approximation of the target surrogate signal y is defined as $\hat{y}≔M\left(X\right)$ . Subsequent to model inference, the estimated time-series $\hat{y}$ is frequently subjected to post-processing in order to enhance the clarity and centering of the characteristic peak. This can be achieved by utilizing a low-pass filter or a moving average.

Finally, a classical peak detection algorithm is applied, with temporal and magnitude thresholds that have been optimized for the purpose of extracting heartbeats. Furthermore, the algorithm may also employ adaptive thresholds. Formally, the set of J-peaks $\hat{P}=\left\{p\in R\right\}$ is extracted from the approximated surrogate signal $\hat{y}$ using a peak detection algorithm. The individual timestamps of the heartbeats, denoted by p , constitute the elements of the set.

We hypothesize that the state-of-the-art method of binary event-hot encoding of heartbeats may not be optimal for J-peak detection, resulting in imprecise event detection. The surrogate approximation $\hat{y}$ may be skewed after the low-pass post-processing, which could lead to inaccurate peak detection. Consequently, it is postulated that the encoding of heartbeats, designated as “kernels” in this paper, exerts a significant influence on the efficacy of subsequent J-peak detection.

To the best of our knowledge, no existing literature addresses the optimal kernel for the encoding of J-peaks with the aim of improving the precision of J-peak detection. A more general literature review, not limited to BCG data, revealed a single similar approach to event extraction from time-series data using deep learning (Azib et al., 2023). This work provides a theoretical framework for event detection in time-series for interval-based events, which was validated on fraud events. In this paper, we propose multiple encodings of heartbeats for generating the surrogate signal and empirically evaluate them with the aim of optimizing the J-peak detection $\hat{P}$ by aiding the model to learn $M:X\mapsto \hat{y}$ . The method will be introduced in detail in Section 4.2.

## 4 Materials and methods

### 4.1 Data acquisition

The dataset comprises both the BCG and ECG, which were collected from 11 participants over a period of approximately 8 h during 17 nights of sleep. The data were collected as part of the Virtual Sleep Lab project, as detailed in Kranzinger et al. (2023). The electrocardiogram (ECG) was recorded using the *BrainAmp* Standard Amplifier (Brain Products GmbH, Germany), a laboratory-standard device known for high-quality recordings. The BCG was measured using an inertial measurement unit (IMU) with a 16-bit resolution (0.06 mg/LSB), and was mounted within the mattress centrally underneath the expected position of the subjects’ chests. The accelerations in three dimensions were sampled at a rate of 1,000 Hz. Subsequently, the signal was interpolated with a cubic spline and resampled at 64 Hz. In total, more than 140 h were measured, with an average interbeat interval (IBI) of 0.92 s.

### 4.2 Proposed method

**FIGURE 2:**
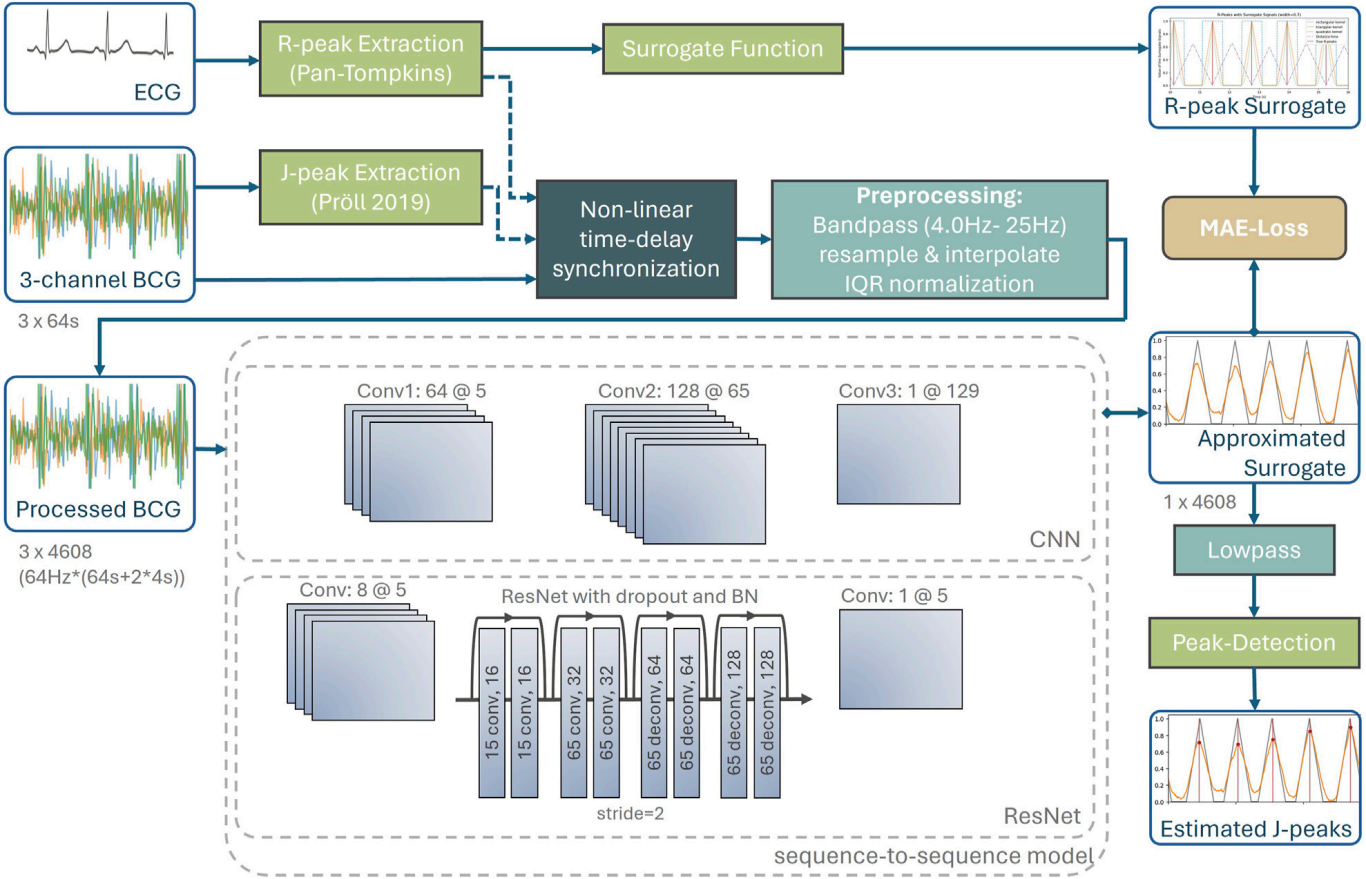
Complete processing pipeline of the proposed method, including synchronization with ground truth heartbeats accessed from the ECG, preprocessing, neural networks as well as post-processing to extract the J-peaks from the approximated surrogate signal.

For BCG, no ground truth J-peaks annotations exist. Therefore, the reference heartbeats from the simultaneous ECG measurement are accessed. The evaluated event detectors are quantified by comparing the estimated J-peaks with the related reference R-peaks using multiple evaluation metrics. Figure 2 depicts the complete processing pipeline of the proposed method. Within this section, each part of the pipeline, from high-precision data synchronization to the final extraction of the estimated J-peaks, is explained in detail.

#### 4.2.1 High-precision data synchronization

A supervised machine learning setting requires ground truth heartbeat events that can be detected from ECG or the less accurate photoplethysmogram (PPG). According to the literature, the RJ-intervals, i.e., the time delay between R-peak and J-peak, vary typically between 180 ms and 240 ms. They may depend on certain factors, such as respiration, however, their changes are slow (Casanella et al., 2012).

In supervised machine learning setups for the J-peak detection utilizing R-peaks extracted from a synchronized ECG as target events, this would lead to varying RJ-intervals along the measurement. A practical approach would be to assume a constant RJ-interval per measurement as correct time-delay. However, the variation of 60 ms of the RJ-interval might lead to a suboptimal heart peak detection precision.

In this work, this issue is addressed by applying a non-linear time-delay synchronization for event-based time-series data (Schranz et al., 2024) between J-peaks and their corresponding R-peaks such that the slowly varying RJ-intervals can be approximated to zero for all J-peaks across the measurement.

As a first preprocessing step, a highly accurate time delay estimation between ECG and BCG is performed using the *nearest-advocate* package (Schranz et al., 2024). As this algorithm requires event-based time series data, the R-peaks were extracted from the ECG and the J-peaks from the BCG using a digital signal processing approach (Pröll et al., 2019). This algorithm was used because signal processing methods tend to be more robust on a new dataset, although there are likely to be more precise methods. The *nearest-advocate* package was also used to reduce non-linearities caused by non-linear clock drifts in the measurement systems and physiological variations that cause changes in RJ intervals.

The resulting dataset therefore has a three-dimensional BCG and corresponding R-peak events that are temporally aligned with the target J-peaks. This initial preprocessing step will make subsequent machine learning models more invariant to changing RJ intervals.

#### 4.2.2 Preprocessing

Windows with a duration of 64-s are sampled from the subjects. Each BCG consists of three channels representing the x, y, and *z*-axis of the IMU. A bandpass filter with cutoff frequencies of 4.0 Hz and 25 Hz was applied to each of the three dimensions of the raw BCG signal. According to the standardization approach of (Gomez-Clapers et al., 2014), the high-pass cutoff-frequency should be lower, such as 1.5 Hz, but our hyperparameter optimization has shown that the pipeline yield improved results if signals below 4.0 Hz are omitted. The bandpass-filtered signal is then normalized using the interquartile range, which is less sensitive to outliers than standard z-score normalization.

The *nearest-advocate* time-delay estimation was then applied again within the range of −2 to +2 s to ensure a proper signal quality and synchronicity for BCG and ECG. Windows with a time-delay of 0.1 s or more between R-peaks and preliminary J-peaks were omitted from the training dataset. Although this discarded approximately 32% of the windows, no systematic bias was introduced because the synchronization between ECG and BCG is independent of signal quality and only the latter affects the quality of subsequent model training. All windows were used for the validation dataset.

#### 4.2.3 Heartbeat encoding of the target R-peak

**FIGURE 3:**
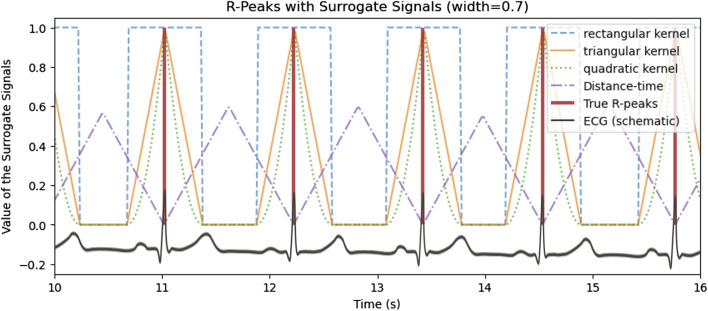
Encoding of the reference heartbeat events in (vertical red lines) with multiple surrogate signals with different encodings.

The core of the proposed approach is the special encoding of heartbeats by a surrogate signal, which is depicted in Figure 3. The reference heartbeats as extracted from an ECG are illustrated in red vertical lines, with four surrogate signals with different encodings. The surrogate signal is the target function that is learned by the neural network. The purpose of the surrogate signal is that the subsequent peak detection is more accurate on the surrogate approximation of the deep learning model.

Therefore, within the scope of the paper, three different kernel shapes, i.e., quadratic, triangular and rectangular, will be empirically evaluated with the aim of finding the most suited kernel for aiding the model to learn $\hat{y}$ . All of these kernels share the properties of being symmetric around the reference heartbeat in the center at a maximum. Note that the rectangular encoding reflects the binary masking of the heartbeat with additional area of interest. Additionally, the distance-time encoding as proposed by (Vijayarangan et al., 2020) for the similar field of R-peak detection in ECG was evaluated. A surrogate signal generated by distance-time encoding has the property, that for any timestamp in y the value represents the distance to the closest heartbeat.

#### 4.2.4 Deep learning models

Two network architectures are evaluated, both implementing a sequence-to-sequence approach, that estimates an equidistant time-series y referred to as surrogate signal.

##### 4.2.4.1 Convolutional neural network

To approximate the surrogate signal, a convolutional neural network (CNN) with three layers of 64, 128, and one channel each is used. The kernels of 5, 65, and 129 are set to become increasingly wider. The model uses the ReLU activation function as a nonlinear mapping between layers and a batch size of 32. The training uses the Adam optimizer with a learning rate of 0.0001 for 40 epochs.

##### 4.2.4.2 Residual Network

Additionally, a Residual Network (ResNet) is applied, as several works in the literature have used a variant of the related ResNet or U-Net architectures (Cathelain et al., 2020; Zhou et al., 2021; Liu et al., 2022). To do this, the initial convolutional layer has 8 channels with a kernel width of 5. Then a ResNet with two convolutional and two deconvolutional residual blocks, each with a step size of 2 was applied. Batch normalization and a 40% dropout were applied between each residual block. Finally, a single-channel convolutional layer with a kernel width of 5 was applied. All other properties are the same as for the CNN.

#### 4.2.5 Post-processing

Since the output of the network is an approximation of the surrogate signal $\hat{y}$ , post-processing is necessary. For this purpose, the model output was smoothed with a second-order low-pass filter with a cut-off frequency of 7.5 Hz. Finally, a peak detection was performed using the *scipy*-package, with the following parameters: distance = 20, height = 0.01, and prominence = 0.1.

### 4.3 Evaluation

The comprehensive method evaluations in (Pröll et al., 2021) only target the accuracy of estimated mean heart rates within 8-s windows. However, the implicit aggregation of heartbeats to mean heart rate limits the applicability for further analyses. For example, the calculation of heart rate variability metrics relies on interbeat intervals (IBI) and reflects a person’s physiological state and health (Shaffer and Ginsberg, 2017). In addition, most sleep stage classification algorithms rely on IBIs as input, i.e., interpolation of the temporal differences between successive heartbeats (Kranzinger et al., 2023).

Since the temporal detection of heartbeats is also important for the subsequent analysis of heartbeats, the detected J-peaks are evaluated using complementary criteria. The following metrics are used for comparison:

1. **HR MAE**: The estimation of heart rates within the full 64-s windows, with deviations reported as mean absolute error (MAE).
2. **HR MAE 8 s**: Estimation of heart rates within a reduced window of 8 s to establish comparability to the proposed methods in of Pröll et al. (2021).
3. **NAd_sym (ms)**: The Nearest-Advocate criterion (Schranz et al., 2024). This quantity is designed to measure the synchronicity between a pair of event-based time series. The resulting value after time-delay correction reflects the average distance between each detected J-peak, and its nearest reference R-peak. The algorithm is applied symmetrically and results are provided in milliseconds (ms). This measure considers only the temporal deviation of detected heart peaks.
4. **IBI MAE (ms)**: MAE between the interbeat interval (IBI) of the detected J-peaks and the reference IBI. Since the precision of the detection is high, the results are given in milliseconds (ms).

In the cross-validation procedure, the individual windows were grouped by subject in order to obtain an unbiased estimator for new subjects. The hyperparameters of the pre-processing, the model, the kernel width, and the post-processing were optimized using a grid search approach.

## 5 Results

**FIGURE 4:**
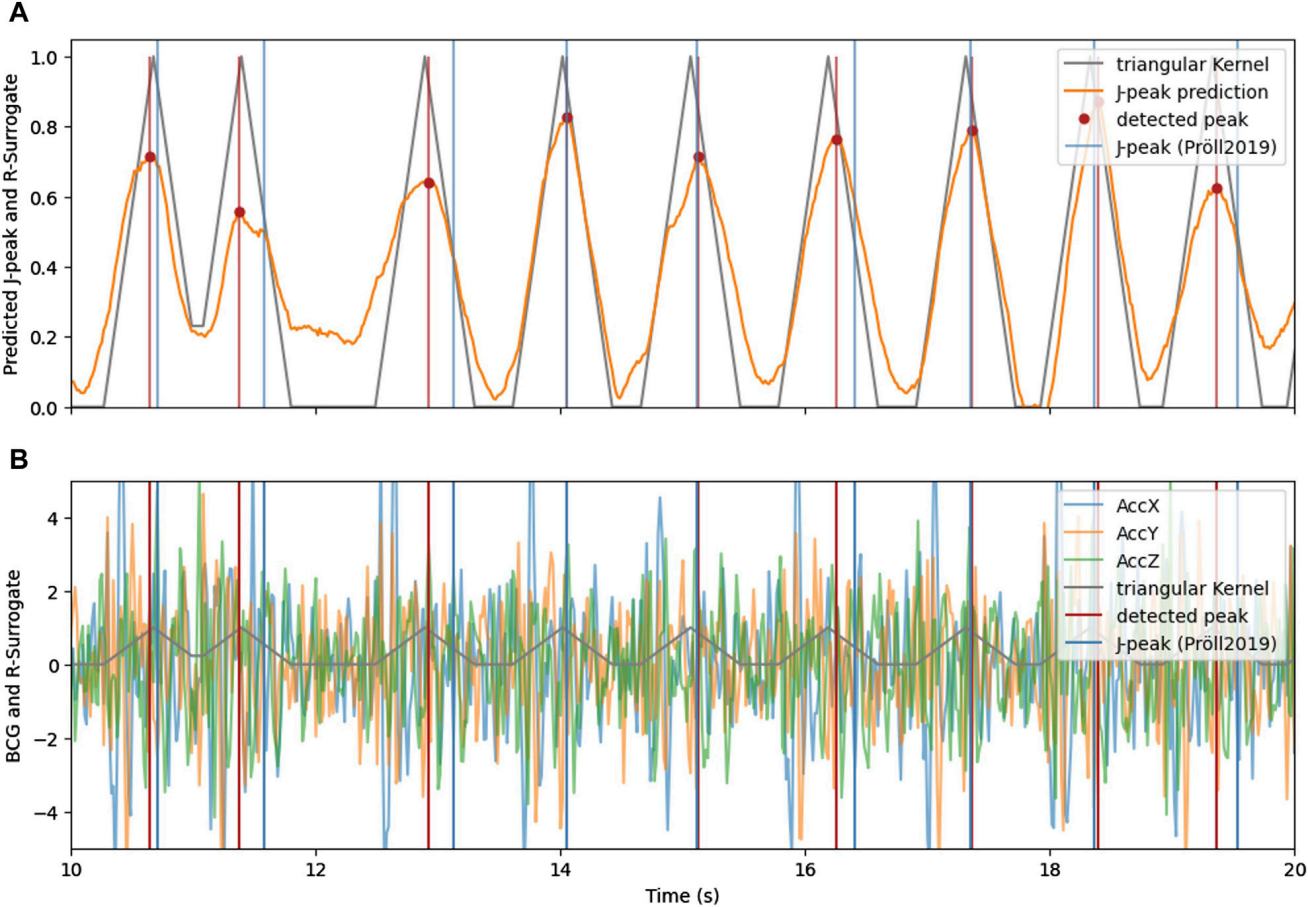
**(A)** The triangular signal (gray) with its approximation by the neural network (orange) and detected heartbeats using peak detection. **(B)** 3-axis BCG with indicated triangular surrogate signal (gray) and the J-peaks as detected by (Pröll et al., 2019) (blue) and with the proposed method and CNN-architecture (red).

Figure 4 shows the intermediate and final results of the proposed J-peak detection pipeline for an exemplary 10-s window. The 3-channel BCG measured by an acceleration sensor from which the heartbeats are to be detected is shown in the lower plot. In both plots, the surrogate signal is illustrated in gray. The heart beats are encoded with triangular kernels, with a center at the exact temporal position of the peak. Heartbeats that are closer together than the kernel width cause interference with super-position, as shown around second 11 in the plot.

The approximation of the surrogate signal (orange) is very similar to the target for clean BCG signals. For noisy episodes in BCG, the approximation remains higher for areas between heart beats, indicating a higher uncertainty of the deep learning estimation. However, even in the noisy area between the 10th and 13th second, the proposed method is able to accurately detect the heart beats.

Table 1 summarizes the results of the experiments. The four measures provided per method are described in Section 4.3. We evaluated our proposed methods against several existing methods, including a state-of-the-art deep learning method of (Pröll et al., 2021). The classical digital signal processing methods were used as implemented in (Pröll et al., 2019) and with default parameters, to facilitate comparability. For Pröll et al. (2021), the best-performing model architecture on their dataset, the Modified CNN-GRUx2, was used and trained on our dataset for 75 epochs. Since this model estimates the mean heartbeat within 8-second windows, only this measure was reported. As this model estimates the mean heartbeat within 8-s windows, only this measure was reported.

**TABLE 1:** Results for each method, with reported mean and standard deviation across subjects. For each evaluation metric, the best result is indicated in bold.

Method/Model	HR MAE	HR MAE 8 s	NAd_sym (ms)	IBI MAE (ms)
Pino et al. (2017)	3.01 ± 2.3	3.83 ± 2.5	65.6 ± 18	57.6 ± 29
Choe and Cho (2017)	4.81 ± 3.9	5.74 ± 4.2	69.6 ± 21	101 ± 66
Brüser et al. (2011)	20.6 ± 4.7	22.5 ± 4.7	95.3 ± 15	215 ± 73
Pröll et al. (2019)	2.32 ± 1.5	3.18 ± 1.6	79 ± 15	78.9 ± 22
Pröll et al. (2021)	—	3.18 ± 0.54	—	—
CNN rectangular kernel	1.46 ± 0.83	2.00 ± 0.89	57.6 ± 7.7	44.2 ± 7.5
CNN triangular kernel	**1.1** ± **0.71**	1.52 ± 0.77	52.4 ± 10	40 ± 8.2
CNN quadratic kernel	1.31 ± 0.74	1.74 ± 0.81	53.8 ± 10	39.8 ± 8.9
CNN Distance Time	1.31 ± 0.88	1.73 ± 0.89	53 ± 9.7	41.4 ± 8.5
ResNet triangular kernel	1.22 ± 0.63	**1.38** ± **0.64**	**48.8** ± **8**	**27.9** ± **7**

The proposed method with a CNN has been evaluated with various kernels (rectangular, triangular, quadratic) as well as the distance-time encoding as proposed by (Vijayarangan et al., 2020) for generating a surrogate signal for ECG. In addition, the most suitable kernel was also evaluated with a ResNet architecture.

The results in Table 1 show the high performance of the proposed method for both for the heart rate estimation [*HR MAE* and *HR MAE 8s*) as well as the precision of the heart peak detection (*NAd_sym (ms)* and *IBI MAE (ms)*]. In particular, the triangular kernel yielded excellent results, with the quadratic kernel and the distance time modelling of the heart peak events being on par.

**FIGURE 5:**
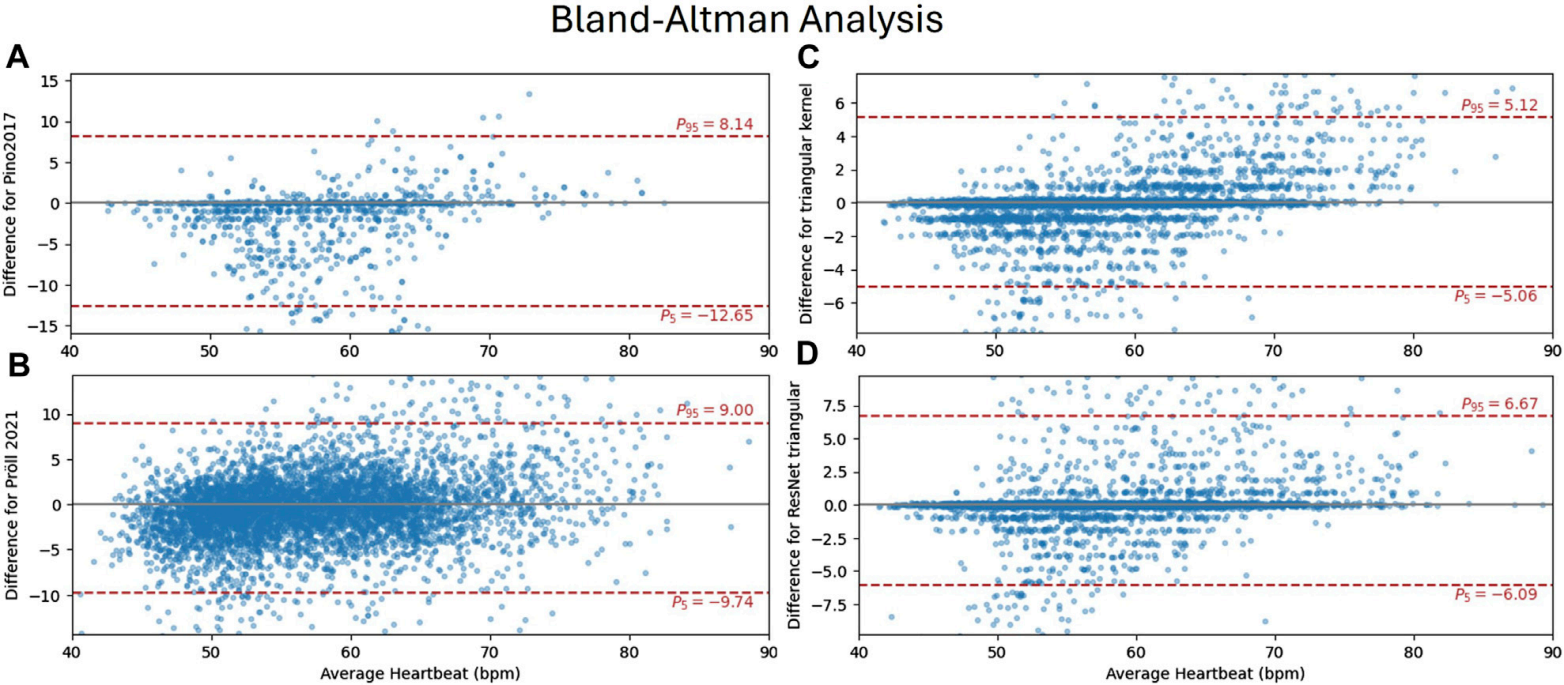
Bland-Altman analysis comparing ground truth heart rates (accessed from ECG) and the methods of (Pino et al., 2017) **(A)** (Pröll et al., 2021), **(B)** and the proposed CNN **(C)** and ResNet **(D)** with triangular kernel each within 8-s windows. The *y*-axis shows the residual of the estimate $hr_{\mathrm{ECG}}-hr_{\hat{B}\mathrm{CG}}$ in beats per minute (bpm), with limits of agreement (LoA) measuring their deviation.

Figure 5 shows a Bland-Altman analysis for four selected algorithms in subplots a) to d), with their 95%-limit of agreement (LoA) in red (Pino et al., 2017). (a), accurately estimates heart rates for a high percentage of windows, as indicated by scatters close to zero (Pröll et al., 2021). (b) is the only method where the quantification of errors resulting from an integer number of heartbeats being incorrectly detected is not visible. This is because this method estimates heart rates directly as a regression task. The proposed methods on the right side (c and d) show very similar distributions, with more outliers for the ResNet (d). This results in a wider LoA, although the MAE of heart rates is lower. For both proposed methods, it can be seen, that multiple outliers are caused by false positives (not detected) events for heart rates around 50 bpm and false negatives (missed events) for higher heart rates.

## 6 Discussion

### 6.1 Method comparison

For each method, the accuracy of heartbeat estimation is better for the full 64-s window than for the reduced 8-s window. The difference of the respective estimations is small, given a reduced interval, by a factor of 8. This can be explained by the implications of falsely detected peaks: Any false positive or false negative peak detection will result in an incorrect number of events within the time range. As the estimated heart rate is calculated as the mean interval between beats, a wider interval is more robust against a missing or incorrectly detected beats. However, the wider interval increases the likelihood of one or more false peaks. Therefore, the difference between *HR MAE* and *HR MAE 8s* is quite small.

For the existing approaches (Pröll et al., 2019; Pröll et al., 2021), the accuracy in terms of heart rates is equivalent. Since Pröll et al. (2021) estimate heart rates for an 8-second window, only this measure can be reported. This method is characterized by a very small standard deviation across subjects, which may indicate an advantage of direct estimation of the target measure. The significant advantage of Pröll’s deep learning method (Pröll et al. 2021) over his classical digital signal processing method, as reported in Pröll et al. (2019), could not be replicated on this dataset within these experiments. This could be explained by an insufficient number of training samples or a more challenging raw BCG signal. There could be explained by a too small number of training samples or a more challenging raw BCG signal.

In contrast, Pino et al. (2017) provides the most accurate detection of J-peaks in terms of *NAd_sym (ms)* and *IBI MAE (ms)*, but has a higher standard deviation and higher error in heart rate estimation. This suggests that the preprocessing method of Pino et al. (2017) may allow for more accurate event detection, but carries a higher risk of false positives or negatives. Furthermore, the transferability to other subjects may be more limited.

The method of Brüser et al. (2011) did not produce the expected results with an MAE of heart rates greater than 20 bpm. We believe that the default parameters were not successful for the given data set. The Bland-Altman analysis (not reported in this work) suggests a plausible range of deviations for heart rates of 50pm , increasing for higher heart rates.

The proposed methods excel for each metric. Regarding the evaluation of heartbeat encodings, the triangular kernel was the most successful for each metric. This indicates a more accurate heartbeat estimation as well as a higher precision of heart peak detection. The hyperparameter optimization suggests a kernel-width of 0.8 s for the triangular kernel and 1.2 s for the quadratic kernel. Furthermore, the inter-subject standard deviation is significantly lower, except for Pröll et al. (2021) concerning the *HR MAE 8s* measure. As expected, the rectangular (binary) encoding of heartbeats yielded solid, but inferior results in comparison to continuous surrogate signals with a single optimum at the heartbeat timestamp.

The evaluation of the more complex ResNet resulted in a higher precision for the J-peak detection, with a mean precision of less than 50 ms. It has been reported in the literature that the subsequent use of a recurrent network such as a GRU or LSTM (long short-term memory) improves the results. Developing the network architecture with a recurrent network or multi-head attention layer is a point for further development.

### 6.2 Kernel evaluation

The results demonstrate that the quality of heartbeat estimation depends significantly on the kernel type utilized to generate the surrogate signal. Therefore, the surrogate signal should be easily learnable by the sequence-to-sequence model and facilitate a precise subsequent peak detection during post-processing. It is hypothesized that the surrogate signal should be continuous and exhibit distinct, well-defined peaks in order to accommodate both properties. Empirical validation has demonstrated that the binary (rectangular) kernel, which yields non-continuous surrogate signal encodings without distinct maxima, is inferior for peak detection compared to other approaches. A visual comparison of the kernels is shown in Figure 3.

Furthermore, the authors hypothesize that the width of the kernel is of importance: On one hand, the width of the kernels should be broad enough to support the target heartbeats also under imprecise measurement or an imperfect dataset. In particular, if the R-peaks are utilized as ground truth heartbeat events, the kernel width must cover also varying RJ-intervals. On the other hand, the width of the kernels, i.e., the support within the surrogate signal, should be constrained, such that points in time not close to peaks have a default value such as zero. This is not the case for Distance Time encoding, where each value represents the time difference to the nearest peak. Moreover, another hypothesis is that the kernel shape should be symmetric in order to improve the learning of the corresponding peak within the signal. However, this was not directly evaluated, rather than indirectly by inference provided by larger kernel width.

Additionally, interferences of two adjacent kernels occur, if the respective peaks are closer together than the kernel width. This issue was particularly evident with the quadratic kernel, which showed optimal performance with a kernel width of 1.2 s, which is greater than the average interbeat interval. Note that the peak of the quadratic kernel is twice as sharp as that of the triangular kernel of the same width in terms of the first derivation. It is still not completely clear why the triangular kernel is superior to the quadratic kernel. The authors hypothesize that there is a trade-off in the kernel width between precision of the peak and inference with adjacent kernel shapes. This does not only involve the already optimized kernel width, but also its shape. It is acknowledged that further research is required to evaluate this trade-off systematically in order to identify the optimal kernel shape for heartbeat encoding.

### 6.3 Intra-subject variability

In the literature, both inter- and intra-subject variability are cited as a major challenge in the analysis of BCG signals (Choe and Cho, 2017; Sadek and Abdulrazak, 2021). In all the results above, the cross-validation was grouped by participants to access the inter-subject variability.

To analyze the within-subject variability only, the effects of classical cross-validation without grouping by subject were analyzed. It was found that the validation error is only about 10% (instead of 80%) higher than the training error. This indicates that the extraction of R-peaks is subject to high interpersonal variability and could be generalized very well across time intervals from the same subject. It is therefore expected that an increase in the number of subjects from the current 11 (with a total of 17 nights) will significantly improve the quality of the model. Alternatively, this work can support the development of data augmentation methods to improve model performance in an original measurement without additional subjects.

### 6.4 Limitations and further work

A limitation of this work is that the limitation of the dataset that was acquired from only eleven participants and over 17 nights. Section 6.3 suggests a much very high intra-subject generalization, however, the inter-subject generalization is significantly lower. In future work, a more comprehensive as well as open dataset will be utilized to get more robust results and to establish a more rigorous method comparison. Furthermore, data augmentation will be employed with the aim to improve the inter-subject generalization. Regarding the dataset, the anthropometries of the subjects should be critically reviewed, especially considering diversity and fit to potential target user groups.

Another limitation of the used dataset was the need for non-linear time synchronization due to temporal issues in the ballistocardiogram data acquisition. To solve this issue, a non-linear time synchronization as suggested in Schranz et al. (2024) was conducted between R-peaks from ECG and preliminarily extracted J-peaks from BCG using the method of Pröll et al. (2019). As the RJ-interval is non-zero and changing slowly (Casanella et al., 2012), the non-linear time synchronization may have compensated the varying time-delay between R-peak and subsequent J-peak. Therefore, this preprocessing step that was employed with the intention to compensate a measurement issue might have improved the suitability of using R-peaks as ground truth events for training a supervised neural network for J-peak extraction. The varying RJ-intervals are typically considered as constant in current literature, or this property is noted as open issue. In order to answer this research question, a very precisely synchronized dataset is required, which further increases the interest in continuing the current work on a larger and open dataset with a dedicated research focus on the preprocessing of BCG data.

Another discussion point for future research is the superiority of the triangular kernel over the quadratic kernel. Here, more experiments should be conducted to systematically evaluate the trade-off mentioned in section 6.2, in order to identify the optimal kernel shape for heartbeat encoding.

## 7 Conclusion

In this work, a method for improved heartbeat detection in BCG is proposed. This method uses various kernel shapes to generate surrogate signals that encode the discrete heartbeat events. Using deep learning models in a sequence-to-sequence setting, this surrogate signal is approximated, allowing a more precise J-peaks extraction in the subsequent peak detection. To the best of our knowledge, this is the first time temporal events are encoded with kernels to enable an improved event detection using a regression-based sequence-to-sequence model. Moreover, this work conducted the first comparison of various event encodings for event detection using deep learning.

The evaluation of different kernel shapes showed, that the simple triangular kernel provided the best surrogate signal to extract J-Peaks with a high precision. Using the proposed method, the MAE of the estimated heart rate was 1.1 s within 64-s and 1.52 s for an 8-s window, halving the precision of the best evaluated existing approach. Compared to a CNN architecture, ResNet architecture improved the accuracy of heartbeat detection, with a mean accuracy of less than 50 ms.

The findings may provide a foundation for enhanced health monitoring during sleep, including comprehensive heart rate variability analysis and sleep stage classification. This research further substantiates the potential of ballistocardiogram sensor technology for unobtrusive and cost-effective health monitoring.

There are several options for future development of the proposed method. Bland-Altman analysis provides quantified estimates. Optimizing the mean HR as an additional target measure with a hybrid loss in the deep learning model training could further improve the heart rate estimation. The overall estimation accuracy could be further improved by adding a recurrent layer after the CNN respectively ResNet architecture layers. A larger or openly available dataset could be used to perform a rigorous comparison of methods, including additional deep learning approaches. This could reduce the high inter-subject variability of the current evaluation. Furthermore, the use of data augmentation methods is well suited to address both intra- and inter-subject variability.

In conclusion, the proposed triangular and quadratic kernels for generating a surrogate signal to be approximated is a novelty and showed significant improvements for J-peak detection in BCG compared to existing solutions. This undermines our initial hypothesis that the design of the surrogate signals for target measures has a significant impact on the quality of the output. This approach can also provide a general solution for applying deep learning models, especially in the sequence-to-sequence setting, for event detection in univariate or multivariate time series data.

## Data Availability

The raw data supporting the conclusions of this article will be made available by the authors, without undue reservation.
